# Age-period-cohort analysis of syphilis epidemics in Eastern China, China, 2005–2024

**DOI:** 10.3389/fpubh.2025.1606491

**Published:** 2025-07-07

**Authors:** Zheyuan Ding, Shuangqing Wang, Junjie Li, Haocheng Wu, Qinbao Lu, Xinyi Wang, Tianyin Fu, Kui Liu, Chen Wu

**Affiliations:** ^1^Department of Public Health Surveillance and Advisory, Zhejiang Provincial Center for Disease Control and Prevention, Hangzhou, Zhejiang Province, China; ^2^Department of Infectious Disease Control, Quzhou Center for Disease Control and Prevention, Quzhou, Zhejiang Province, China

**Keywords:** syphilis, reported incidence, age-period-cohort model, Eastern China, cohort effect

## Abstract

**Background:**

Syphilis remains one of the serious public health challenges in China and worldwide. This study aims to assess the potential independent risks associated with age, period, and birth cohort for the reported incidence of syphilis in Eastern China.

**Methods:**

Data on all syphilis cases from 2005 to 2024 in Zhejiang Province in Eastern China were collected from the China Information System for Disease Control and Prevention. The Age-Period-Cohort (APC) model was used to analyze the effect coefficients, which were then converted into relative risks (RRs).

**Results:**

From 2005 to 2024, a total of 617,097 syphilis cases were reported in Zhejiang Province. The reported incidence of syphilis decreased by an average of −3.53% per year across all age groups (95% CI: −4.34, −2.70%). The age effect showed that the highest reported incidence was among individuals aged 20–24 years, with a relatively high rate also observed in those aged 60 years and above. The period effect indicated that compared with the reference group of 2010–2014 with the highest reported incidence risk, the risk in 2020–2024 was the lowest (RR = 0.48; 95% CI: 0.43, 0.54). The cohort effect shows that the risk decreased in later birth cohorts, with the highest reported incidence risk in the birth cohort of 1960–1964 (RR = 1.30; 95% CI: 1.08, 1.57) and the lowest risk in the birth cohort of 2020–2024 (RR = 0.00; 95% CI: 0.00, 0.18).

**Conclusion:**

The reported incidence of syphilis in Zhejiang Province has shown an overall downward trend. The implementation of national syphilis control programs has achieved significant results. There is a need to strengthen the management of late-stage syphilis among older adults and enhance syphilis prevention and control efforts among adolescents.

## Introduction

Syphilis is a sexually transmitted infection (STI) caused by *Treponema pallidum* (1). It can also be transmitted from mother to fetus, called congenital syphilis (2). Globally, the number of prevalent syphilis cases was estimated to be 49.71 million in 2019, marking a 60.83% increase from 1990, creating an significant global burden (3). In China, the incidence of syphilis has risen significantly over the past decades, with an average annual growth rate of 11.88% between 2004 and 2019 (4). In June 2010, the Ministry of Health of China launched the National Syphilis Prevention and Control Program (2010–2020) (National Syphilis Control Program), which aimed to strengthen the prevention and control of syphilis and effectively curb its spread (5). The program set specific control targets for primary and secondary syphilis, as well as congenital syphilis. In 2011, a further national plan was released, specifically targeting the Preventing Mother-to-Child Transmission of HIV, syphilis, and hepatitis B (PMTCT) (6). These programs have achieved significant results. Between 2014 and 2019, the reported incidence of primary and secondary syphilis, as well as congenital syphilis, in China decreased annually by 10.95 and 26.16%, respectively (7). Meanwhile, the incidence risk of syphilis in China varies across different regions. Studies have shown that there is a certain temporal and spatial clustering of syphilis cases in the southeastern coastal areas (8). These regions still face significant challenges in syphilis prevention and control.

As a vital constituent province of the eastern coast of China, Zhejiang Province has a highly developed economy and significant population mobility (9). Over the past decade, Zhejiang Province has consistently ranked among the top in China in terms of the reported incidence of syphilis (4, 7). Moreover, for many years, syphilis has topped the list of notifiable infectious diseases in Zhejiang Province (10), highlighting its significant public health impact. Syphilis prevention and control has always been an important task in the public health domain of Zhejiang Province. Previous studies (10–12) have described the epidemic trends, spatial distribution, and population characteristics of syphilis in Zhejiang Province, but the analysis and exploration of potential causes have been insufficient. Moreover, cross-sectional studies cannot elucidate the independent effects of age, period, and birth cohort on the reported incidence of syphilis. However, the Age-Period-Cohort (APC) model (13) is capable of doing so. But there were few studies on the application of APC in syphilis in China, as well as in Eastern China.

In this study, we chosen Zhejiang Province as the pilot area, to describe the epidemiological characteristics of syphilis in Eastern China, and employed the APC model to analyze the impact of time, age, and birth cohort on the reported incidence trends of syphilis among different genders and different subtypes of syphilis. It would provide the reliable evidence for the available comprehensive action and help to inform more effective public health interventions for further syphilis control.

## Methods

### Data collection and definitions

Data on syphilis cases in Zhejiang Province from 2005 to 2024 were obtained from the National Notifiable Infectious Disease Reporting Information System, which is a component of the China Information System for Disease Control and Prevention. Syphilis is one of the notifiable infectious diseases in China, and all cases diagnosed by clinicians are required to be reported to this system within 24 h. All cases included in the study were laboratory-confirmed. The variables included in the analysis were sex, age, occupation, time of onset, and syphilis subtype, which was classified into primary syphilis, secondary syphilis, tertiary syphilis, latent syphilis, and congenital syphilis. All notified syphilis cases were diagnosed and subtyped in accordance with the Chinese National Diagnostic Criteria and management of Syphilis (GB 15974—1995) (14) before 2007, the National Diagnostic Criteria for Syphilis (WS 273—2007) (15) from 2007 to 2017, and the National Diagnostic Criteria for Syphilis (WS 273—2018) after 2018 (16). Primary syphilis, secondary syphilis, and early latent syphilis (infection duration less than 2 years) are classified as early syphilis, while tertiary syphilis and late latent syphilis (infection duration exceeding 2 years) are classified as late syphilis (16, 17). Since latent syphilis is asymptomatic and without signs, it must be detected through screening tests, making it difficult to determine whether it is a new infection or a past infection. Therefore, in this study, the incidence of primary and secondary syphilis is used to reflect the situation of new infections.

### Statistical analysis

Epidemiological characteristics of syphilis in Zhejiang Province from 2005 to 2024 were analyzed. APC model was used to evaluate the age, period, and cohort effects of trends in the reported incidence of syphilis among the entire population, as well as separately among males and females. Besides, stratification analyses of APC model were also conducted in primary syphilis, secondary syphilis, tertiary syphilis and latent syphilis. In this study, we divided age into 18 groups, with each group spanning 5 years (0–4, 5–9, …, 80–84, 85–89). The periods were divided into four intervals (2005–2009, 2010–2014, 2015–2019, and 2020–2024), with the middle period (2010–2014) as the reference. The birth cohorts were determined by subtracting age from the periods, resulting in 21 cohorts (1920–1924, 1925–1929, …, 2015–2019, 2020–2024), with the middle cohort group (1970–1974) as the reference. The APC model is a type of generalized linear model, with the basic expression as follows:

$$\log R_{\mathrm{ijk}}=\log(E_{\mathrm{ij}}/N_{\mathrm{ij}})=\mu +\alpha \times {\mathrm{Age}}_{i}+\beta \times {\text{Period}}_{j}+\gamma \times {\text{Cohort}}_{k}+\varepsilon$$

*R*_ijk_ represents the reported incidence of syphilis in age group i, period j, and birth cohort k; *E*_ij_ and *N*_ij_ represent the expected number of syphilis cases and population at risk in age group i, period j, respectively; μ is the reference level of disease risk; Age i represents the effect of the age group i; Period j represents the effect of the period j; Cohort k represents the effect of the birth cohort k associated with the age group i and the period j. Parameters *β* and *γ* were converted exponentially to represent the relative risk (RR) of a particular period and birth cohort. The significance of the estimable parameters was evaluated using the two-sided Wald *χ*^2^ test. APC analyses were performed using a web tool from the National Cancer Institute of the United States^1^ and R software (version 4.3.0; R Core Team, Vienna, Austria) (13).

## Results

### General characteristics of syphilis in Zhejiang Province during study period

Between 2005 and 2024, a total of 617,097 cases of syphilis were reported in Zhejiang Province, including 129,698 cases of primary syphilis, 104,513 cases of secondary syphilis, 3,731 cases of tertiary syphilis, 7,556 cases of congenital syphilis, and 371,598 cases of latent syphilis. The number of female cases was higher than that of male cases. However, for primary syphilis, congenital syphilis, and especially tertiary syphilis, the number of male cases exceeded that of female cases. The majority cases were in the age group of 15–39 years old, accounting for 47.5% of the total cases, followed by the age group of 40–59 years old, which accounted for 31.7%. For tertiary syphilis, the proportion of cases in individuals over 40 years old was relatively higher. Congenital syphilis mainly affected children under 14 years old. Except for congenital syphilis, the other types of syphilis were predominantly found among farmers, who accounted for approximately 40% of the cases (Table 1).

**Table 1:** Epidemiological characteristics of syphilis in Zhejiang Province from 2005 to 2024.

Variables	Syphilis	Primary syphilis	Secondary syphilis	Tertiary syphilis	Congenital syphilis	Latent syphilis
Total, *n* (%)	617,097(100)	129,698(21.0)	104,513(16.9)	3,731(0.6)	7,556(1.2)	371,598(60.2)
Sex, *n* (%)						
Male	288,740(46.8)	66,629(51.4)	51,156(48.9)	2,593(69.5)	4,162(55.1)	164,199(44.2)
Female	328,357(53.2)	63,069(48.6)	53,357(51.1)	1,138(30.5)	3,394(44.9)	207,399(55.8)
Age group (years), *n* (%)						
0–14	8,662(1.4)	347(0.3)	211(0.2)	4(0.1)	7,515(99.5)	585(0.2)
15–39	293,216(47.5)	73,659(56.8)	60,826(58.2)	792(21.2)	30(0.4)	157,909(42.5)
40–59	195,528(31.7)	40,352(31.1)	33,189(31.8)	1,628(43.6)	4(0.1)	120,355(32.4)
≥60	119,690(19.4)	15,340(11.8)	10,287(9.8)	1,307(35.0)	7(0.1)	92,749(25.0)
Population classification, *n* (%)						
Farmer	247,378(40.1)	51,945(40.1)	38,954(37.3)	1,531(41.0)	23(0.3)	154,925(41.7)
Houseworkers or unemployed	100,955(16.4)	17,128(13.2)	15,420(14.8)	598(16.0)	9(0.1)	67,800(18.2)
Industrial workers	62,960(10.2)	16,881(13.0)	14,259(13.6)	336(9.0)	3(0.0)	31,481(8.5)
Commercial service worker	51,669(8.4)	9,805(7.6)	9,666(9.2)	292(7.8)	6(0.1)	31,900(8.6)
Retiree	24,754(4.0)	2,687(2.1)	1910(1.8)	308(8.3)	4(0.1)	19,845(5.3)
Migrant worker	20,855(3.4)	8,114(6.3)	4,512(4.3)	83(2.2)	2(0.0)	8,144(2.2)
Preschool children	7,613(1.2)	261(0.2)	104(0.1)	2(0.1)	6,900(91.3)	346(0.1)
Others	100,912(16.4)	22,877(17.6)	19,688(18.8)	581(15.6)	609(8.1)	57,157(15.4)

### Results of estimated annual percentage change (EAPC) for syphilis

**Figure 1:**
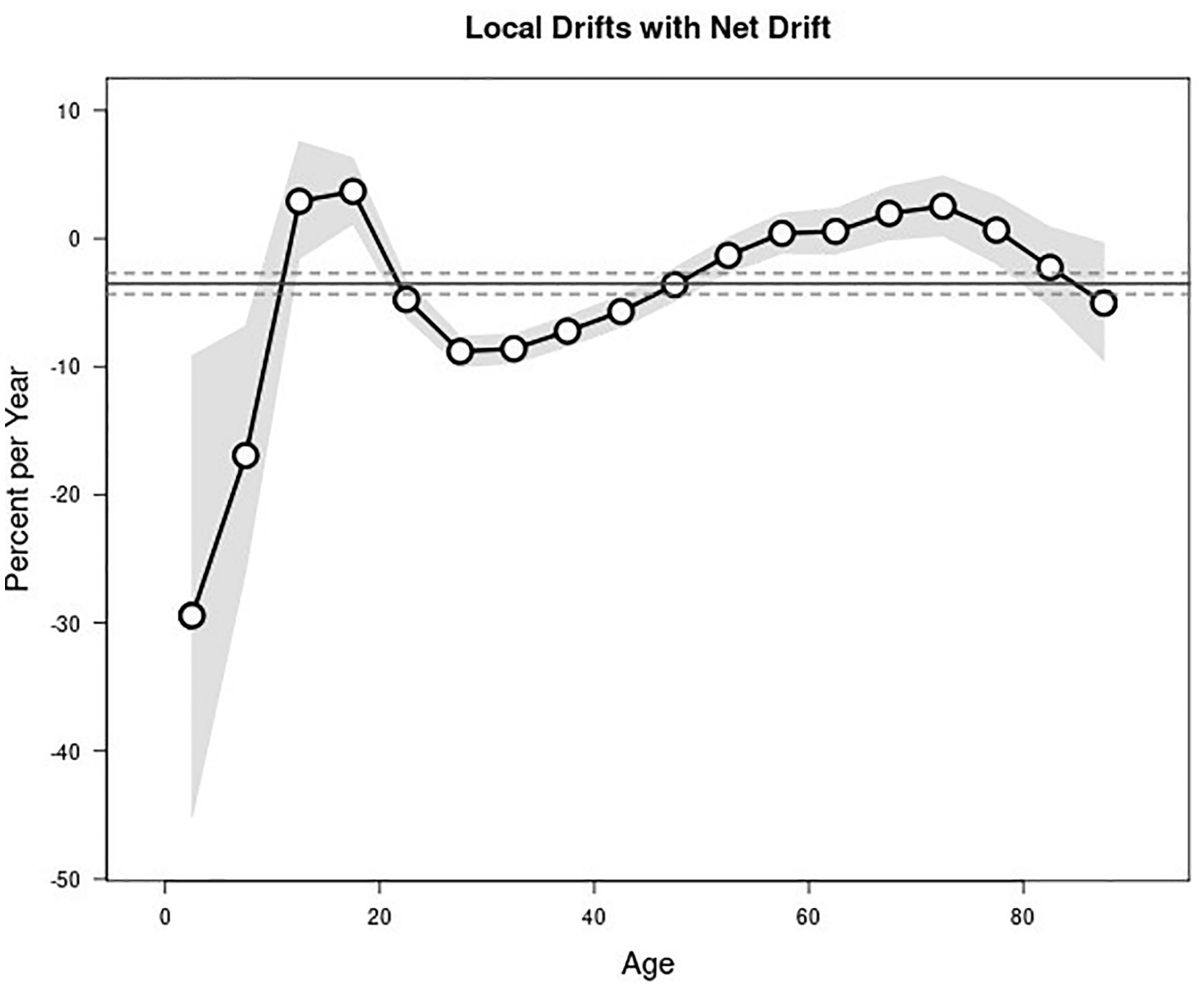
Estimated annual percentage change of reported incidence of syphilis in each age groups in Zhejiang province.

During the study period, the EAPC of the reported incidence of syphilis among all age groups in Zhejiang Province was −3.53% (95% CI: −4.34, −2.70%). The EAPC varied significantly across different age groups. A downward trend was observed in the 0–9 and 20–49 age groups, with the notable reductions in the 0–4 and 5–9 age groups, having EAPCs of −29.43% (95% CI: −45.19, −9.14%) and −16.94% (95% CI: −25.95, −6.83%), respectively. Additionally, the 25–29, 30–34, and 35–39 age groups also experienced significant declines, with EAPCs of −8.81% (95% CI: −10.00, −7.61%), −8.61% (95% CI: −9.74, −7.46%), and −7.23% (95% CI, −8.42, −6.03%), respectively. However, the 15–19 age group showed an upward trend, with an EAPC of 3.68% (95% CI, 1.14, 6.28%) (Figure 1).

The reported incidence of syphilis declined by an average of 3.00% (95% CI: −3.78, −2.21%) per year among males and 3.08% (95% CI: −4.07, −2.07%) per year among females. Among the 0–9 and 35–49 age groups, the decline was greater in males than in females, while among the 20–34 age group, the decline was greater in females than in males (Supplementary Figure S1).

The further stratified analysis showed that the reported incidence of primary and secondary syphilis declined across all age groups. The EAPCs were −14.67% (95% CI: −17.97, −11.23%) for primary syphilis and −8.43% (95% CI: −9.82, −7.03%) for secondary syphilis, with a greater decline observed in females than in males. In contrast, latent syphilis incidence increased slightly, with an EAPC of 1.97% (95% CI: 0.80, 3.16%), which were higher in males than in females. The EAPC for tertiary syphilis was not statistically significant. Among individuals aged ≥15 years, the reported incidence of primary syphilis decreased across all age groups, with a remarkable decline in females. For secondary syphilis, the reported incidence decreased across all age groups of individuals aged ≥20 years, with a greater decline in females aged 20–49 years. The reported incidence of tertiary syphilis increased in individuals aged ≥70 years. For latent syphilis, the reported incidence increased in individuals aged 10–14 years (EAPC: 12.36, 95% CI: 1.98, 23.79%) and 15–19 years (EAPC: 9.91, 95% CI: 7.25, 12.63%), and showed a slight increase in those aged 45–79 years, while it decreased in individuals aged 25–39 years (Supplementary Tables S1–S4).

### Age effect

**Figure 2:**
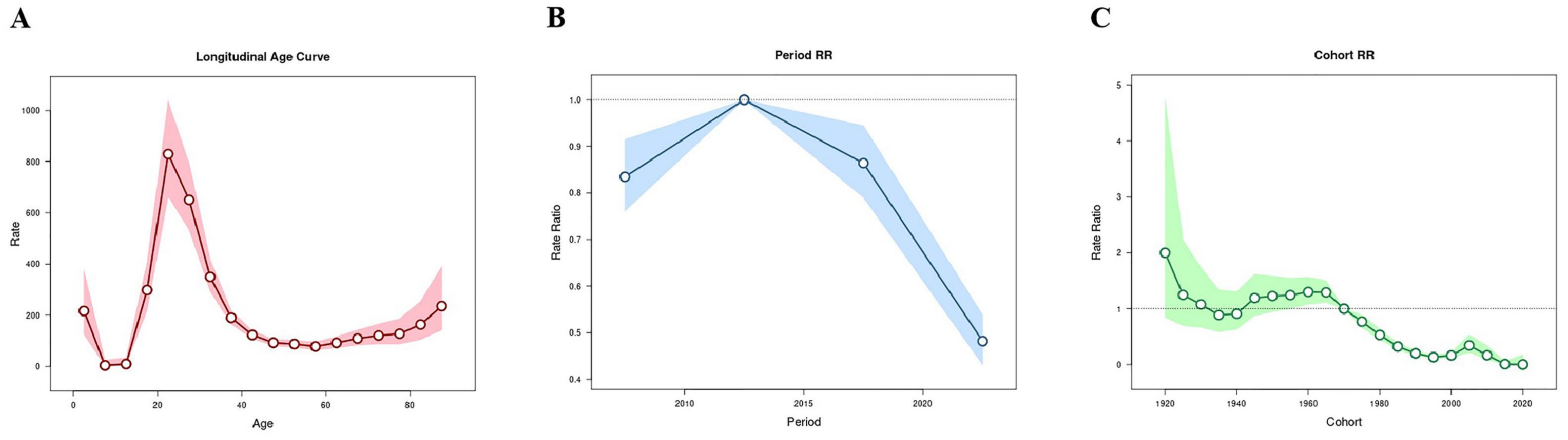
Age-period-cohort effects on the reported incidence of syphilis in Zhejiang Province. **(A)** Age effect. **(B)** Period effect. **(C)** Cohort effect. The colored area represented the 95% confidence interval (CI).

Given that the absolute value of the net drift exceeded 1%, longitudinal age curves were utilized in this study to delineate the age-specific effects. After adjusting for period and birth cohort influences, the longitudinal age curves revealed distinct patterns in the reported incidence across different age groups. Specifically, the 0–4 age group exhibited a relatively high incidence, which then declined sharply in both the 5–9 and 10–14 age groups, resulting in the lowest reported incidence among these cohorts. After the age of 15, the reported incidence began to rise significantly, peaking in the 20–24 age group. Subsequently, the reported incidence decreased with increasing age, although a slight rebound was observed after the age of 60 (Figure 2).

The age effects in males and females were basically consistent with the overall population effect. It is worth noting that the age group with the highest reported incidence in males was the 25–29 years group, while in females it was the 20–24 years group. Among individuals aged 55–69 years, the reported incidence in males was higher than that in females of the same age group, while in other age groups, the incidence in males was lower than that in females (Supplementary Figures S2, S3).

For primary and secondary syphilis, the reported incidences were relatively higher among individuals aged 15–34 years, with the 20–24 years group having the highest reported incidence. For tertiary syphilis, the reported incidence was relatively higher among individuals aged 80 years and above. For latent syphilis, there was a small peak in the reported incidence among individuals aged 20–34 years, followed by a slight decline, and then a significant increase from the age of 60 years, with the highest rate observed in the 85–89 years group. Overall, in all stages of syphilis, the reported incidence was higher in females than in males among younger age groups, while among older age groups, the reported incidence was higher in males than in females (Supplementary Tables S5–S8).

### Period effect

After controlling for age and cohort variables, the risk on reported incidence of syphilis increased from 2005–2009 to 2010–2014 and then decreased afterward. The lowest risk was observed during 2020–2024 (RR = 0.48; 95% CI: 0.43, 0.54; *p* < 0.001). The analysis results for males and females were generally consistent with the overall population effect (Figure 2 and Supplementary Figures S2, S3).

From 2005–2009 to 2010–2014, the risk on reported incidence of secondary syphilis increased. The risk on reported incidence of primary and secondary syphilis decreased after 2010–2014. For latent syphilis, the risk increased from 2005–2009 to 2015–2019 and then decreased afterward. The period effect for tertiary syphilis was not statistically significant. The results for males and females were generally consistent (Supplementary Tables S5–S8).

### Cohort effect

Overall, the risk on reported incidence of syphilis decreased progressively with more recent birth cohorts. Using the 1970–1974 birth cohort as the reference, cohorts born between 1955 and 1969 exhibited higher risks of syphilis, with the highest risk observed in 1960–1964 birth cohort (RR = 1.30; 95% CI: 1.08, 1.57). In contrast, cohorts born after 1975 had a risk less than 1with a successive declined trend. However, a slight increase was noted in the cohorts born in 2000–2004 and 2005–2009. The lowest risk was in the 2020–2024 cohort (RR = 0.00; 95% CI: 0.00, 0.18). Cohorts born between 1920 and 1954 showed no statistically significant differences. The findings for males and females were largely consistent with the overall population trends (Figure 2 and Supplementary Figures S2, S3).

For primary syphilis, the risk on reported incidence decreased with more recent birth cohorts. The highest risk was observed in the 1920–1924 cohort, while the lowest risk was in the 2015–2019 cohort. For secondary syphilis, a downward trend was also observed. The highest risk was in the 1920–1924 cohort, and the lowest risk was in the 1995–1999 cohort. For tertiary syphilis, cohorts born between 1920 and 1939 had a risk lower than that of the reference cohort (1970–1974), while other cohorts showed no statistically significant differences. For latent syphilis, cohorts born between 1920 and 1964 and between 1985 and 1999 had a risk lower than the reference cohort, while the cohort born in 2005–2009 had a higher risk than the reference cohort. The findings for males and females were generally consistent with the overall population trends (Supplementary Tables S5–S8).

## Discussion

This study conducted a systematic analysis of the reported incidence of syphilis in Zhejiang Province, an eastern coastal region of China, using the APC model. It explored the independent risks associated with age, period, and birth cohort, as well as the potential influencing of factors such as age, gender, socioeconomic changes, syphilis prevention policies, sexual attitudes, and the Coronavirus disease 2019 (COVID-19) pandemic. These findings are expected to play a critical role in promoting the effective implementation of syphilis interventions, thereby reducing the disease burden of syphilis in Eastern China.

Over the past two decades, the reported incidence of syphilis in Zhejiang Province has shown a general downward trend. This differs from the epidemiological trends of syphilis in other countries. Japan has experienced a significant increase in syphilis cases after 2011 (18, 19), while South Korea has seen a more moderate rise (19). The United States also observed an increase in syphilis incidence between 2017 and 2024 (20). In Canada, the incidence of infectious syphilis began to rise steadily in the early 2000s and surged sharply after 2017 (21). These differences may be related to variations in sexual attitudes and behaviors, healthcare accessibility, and public health policies for syphilis among different countries.

Since 2010–2014, there has been a significant reduction in the reported incidence of primary and secondary syphilis in Zhejiang Province. The reported incidence in the 0–9 age group has also declined rapidly over these 20 years, which largely due to the effective control of congenital syphilis. These results implied a downward trend in new syphilis infections and cases of congenital syphilis, which was largely attributed to the successful implementation of the National Syphilis Control Program (5) and PMTCT (6). The effectiveness of these programs has also be proved in other southeastern area in China (22). The specific measures of these two programs include providing universally accessible and free syphilis counseling services, enhancing screening program to promote early diagnosis and treatment, offering standardized, high-quality and accessible diagnostic and treatment services, carrying out extensive health education campaigns, promoting condom use, as well as early detection and intervention for syphilis among pregnant women. Meanwhile, the latent syphilis had shown a slight overall upward trend. Specifically, there was a continuous increase before 2015–2019, followed by a decline from 2020 to 2024. The earlier rise might be mainly due to the standardization of testing and reporting under the National Syphilis Control Program. It encompassed the enhancement of the national syphilis surveillance network, the strengthening of quality control for syphilis testing, and the implementation of active syphilis screening. The subsequent decline was likely attributable to the impact of the COVID-19 pandemic. Although some countries saw a rise in syphilis cases during or after this period (23, 24), in China, STIs such as HIV, gonorrhea, syphilis, hepatitis B, and hepatitis C all presented a decline (25–27). Available evidence demonstrated that the number of reported syphilis cases in China decreased by 17.95% in 2020 and 20.41% in 2021, respectively (28). This phenomenon was not due to decreased diagnostic accuracy or underreporting of syphilis (28). Instead, the pandemic led to a reallocation of medical resources toward COVID-19 control, resulting in weakened or temporarily interrupted routine HIV/STI screening, diagnostic, and treatment services (29). Additionally, restrictions on population mobility were likely to reduce the healthcare-seeking behavior of asymptomatic individuals or cases with mild symptoms that experienced high risk of exposure (30).

The number of reported syphilis cases among sexually active individuals aged 20–34 years was relatively high, especially for primary and secondary syphilis, which have higher incidence in this population. Compared to males, females had a higher risk, and the peak age group was younger. This might be due to differences between males and females in biological risk of STI infection, clinical manifestations, and sexual behavior characteristics (31), causing STIs more easily transmitted from males to females (32). Moreover, this specific age group was the childbearing period for females, and prenatal screening increased the detection of syphilis. The reported incidence of syphilis in the 15–19 age group showed an upward trend over the past 20 years, with a rapid increase in the reported incidence of latent syphilis in the 10–19 age group. This indicated that syphilis infection in Zhejiang Province was beginning to affect younger populations. This is consistent with the increasing trend of HIV infections among young students in China (33). This might be because contemporary youth were more physiologically and psychologically precocious, with related liberal sexual attitudes (34). Additionally, China tended to adopt abstinence-based education strategies, which could result in adolescents with insufficient knowledge about STIs (35), lack of self-protection awareness, and a higher likelihood of engaging in risky sexual behaviors (36). Thus, comprehensive sex education programs should be introduced for young people, along with support from families and society, as well as sexual and reproductive health services, to reduce the risk of syphilis transmission (34).

Tertiary syphilis was associated with severe complications affecting the nervous system, cardiovascular system, eyes, and other organs (37), which could significantly impair quality of life. Meanwhile, latent syphilis, with its large case base and comprising over half of all syphilis cases, also contributed substantially to the overall disease burden. This study observed an increase in tertiary syphilis among individuals aged 70 and above. Moreover, the reported incidence of syphilis, particularly tertiary and latent syphilis, were found to be higher in the older adult population. These findings were consistent with other studies in Guangdong Province (38), Shandong Province (39), and Japan (40). Among older adults, especially in rural areas, commercial sex and high-risk sexual behaviors were relatively common while condom use was infrequent (41, 42). Compared to younger individuals, older adults generally had lower awareness of the risks associated with STIs and experienced higher levels of stigma (38). Besides, physiological changes during aging, such as increased fragility of the vaginal mucosa and micro-abrasions after intercourse, could elevate the risk of STIs (43). Additionally, older adults often pay less attention to infections, leading to delayed medical visits (44) and interrupt treatment. This frequently results in early-stage syphilis progressing to tertiary syphilis. Besides, given that older adults were more likely than younger individuals to be hospitalized for underlying diseases, the higher reported incidence of latent syphilis among older adults may be attributed to the increased likelihood of syphilis screening and detection during hospitalization. Therefore, it was a need to strengthen sexual health education among older adults, disseminate knowledge about STIs, advocate the use of condoms, and eliminate feelings of shame and inferiority. Simultaneously, sufficient medical services and effective health support should be provided for older adults, including the establishment of geriatric sexual health clinics, and STI screening for high-risk older adult groups to reduce the risk of syphilis and other STIs among the older adult population.

As birth cohorts progressed, the reported risk of syphilis, particularly for primary and secondary syphilis, declined. This trend was largely attributed to a series of syphilis control campaigns in China. In the nascent years of New China, following its founding in 1949, syphilis emerged as one of the most pressing public health challenges (45). Between the early 1950s and 1964, China initiated its first comprehensive nationwide campaign against syphilis (46). This multifaceted effort encompassed the closure of brothels, the implementation of extensive health education programs, widespread screening initiatives, and the provision of free treatment for those infected. By 1964, these concerted actions had basically eradicated syphilis across the nation (46, 47). Subsequently, syphilis virtually disappeared from public view. However, in the early 1980s, with the comprehensive implementation of economic reform and opening-up policies, significant changes occurred in the socio-economic and life environment, as well as in people’s attitudes toward sex. These changes included increased economic levels, greater population mobility, commercial sex, and extramarital sex, all of which contributed to the resurgence of syphilis (48, 49). In response, China launched its second nationwide syphilis control campaign (49), including establishing the Chinese National Center for STD (Sexually Transmitted Disease) Control in 1986 and the national STD surveillance system in 1987. Health education and behavior change initiatives, with a focus on promoting condom use, have been prioritized as key public health strategies to address the syphilis epidemic (46). Since the late 1990s, the annual growth rate of syphilis has slowed down but remains high (50), particularly with increasing prevalence among high-risk populations such as female sex workers and men who have sex with men (51, 52). According to our findings, cohorts born before 1970–1974 exhibited a continuous increase in the reported risk of latent syphilis. Additionally, the reported incidence of tertiary syphilis in the 1970–1974 birth cohort was higher than that in cohorts born before 1935–1939. These patterns might be attributed to the impact of these historical junctures, as well as the development of the internet, which has facilitated the use of social software to seek sexual partners and thereby increased the difficulty of syphilis prevention and control. The study also observed a slight increase in cohorts born in 2000–2004 and 2005–2009, likely due to the intensified syphilis testing efforts under the National Syphilis Control Program implemented after 2010.

### Limitations

Some limitations also should be mentioned. First, our data was derived from a passive surveillance network, which might be subject to reporting bias. Secondly, this study lacked a specific analysis of congenital syphilis. Since the potential population affected by congenital syphilis were neonates rather than the general population, it was not feasible to conduct relevant analyses using the APC model. Third, as an emerging intervention for syphilis prevention, pre-exposure prophylaxis for HIV (PrEP) was not thoroughly discussed in this study due to the lack of patient-related data.

## Conclusion

Over the past 20 years, the reported incidence of syphilis in Zhejiang Province showed a general downward trend. Individuals aged 20–34 years were the most susceptible population. However, the high incidence of late-stage syphilis among older adults and the rising risk of syphilis among adolescents were serious issues that warranted attention. These findings could help relevant departments through formulating appropriate policies and implementing targeted prevention and control measures in susceptible individuals.

## Data Availability

The original contributions presented in the study are included in the article/Supplementary material, further inquiries can be directed to the corresponding author.

## Footnotes

^1^ https://analysistools.cancer.gov/apc/

## Supplementary material

SUPPLEMENTARY FIGURE S1 Estimated annual percentage change of reported incidence of syphilis in each age groups and gender in Zhejiang Province.

SUPPLEMENTARY FIGURE S2 Age-period-cohort effects on the reported incidence of syphilis in males in Zhejiang Province.

SUPPLEMENTARY FIGURE S3 Age-period-cohort effects on the reported incidence of syphilis in females in Zhejiang Province.
