# Supplementary figures and images for: Age-period-cohort analysis of syphilis epidemics in Eastern China, China, 2005–2024
Source: Front Public Health. 2025 Jul 7;13:1606491. doi: 10.3389/fpubh.2025.1606491 (PMC12277379; doi:10.3389/fpubh.2025.1606491)

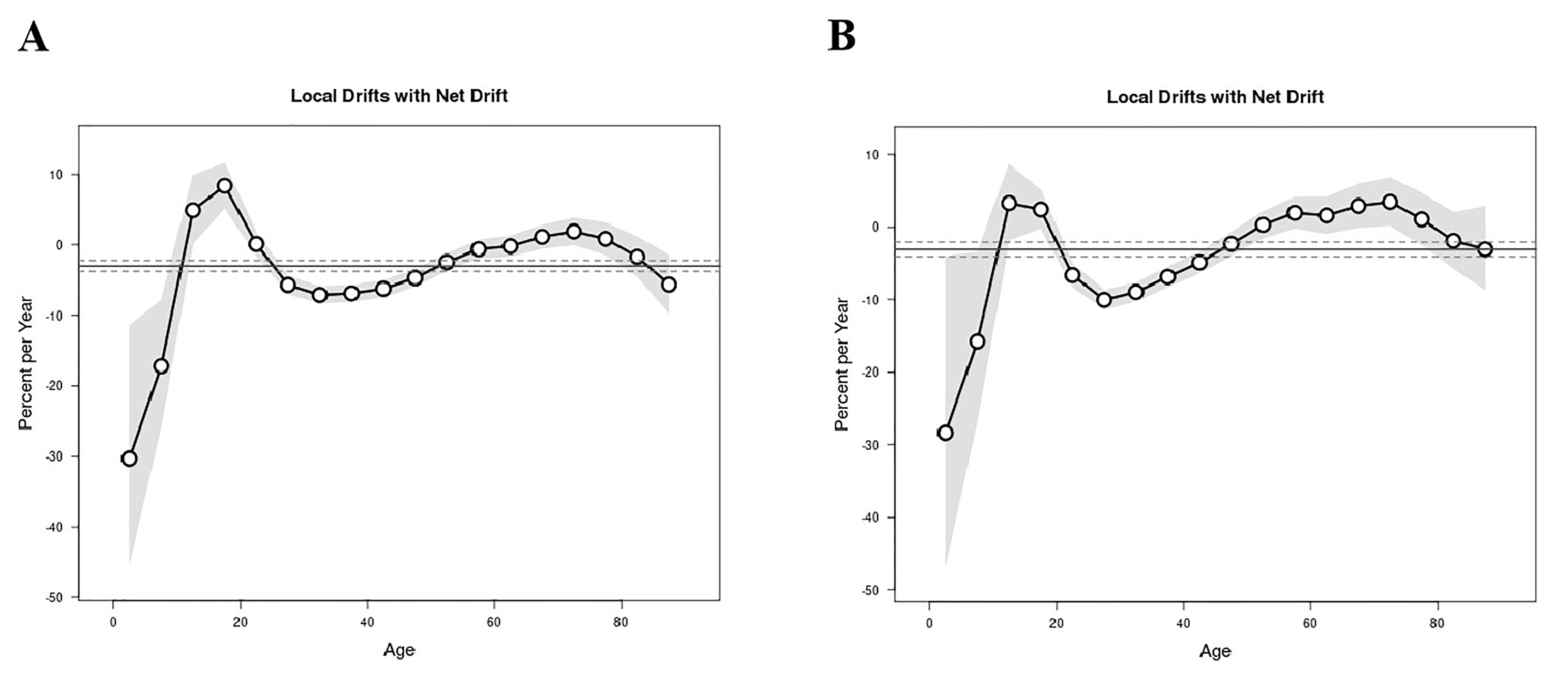

Supplement: SUPPLEMENTARY FIGURE S1 — Estimated annual percentage change of reported incidence of syphilis in each age groups and gender in Zhejiang Province. [file Image_1.JPEG]

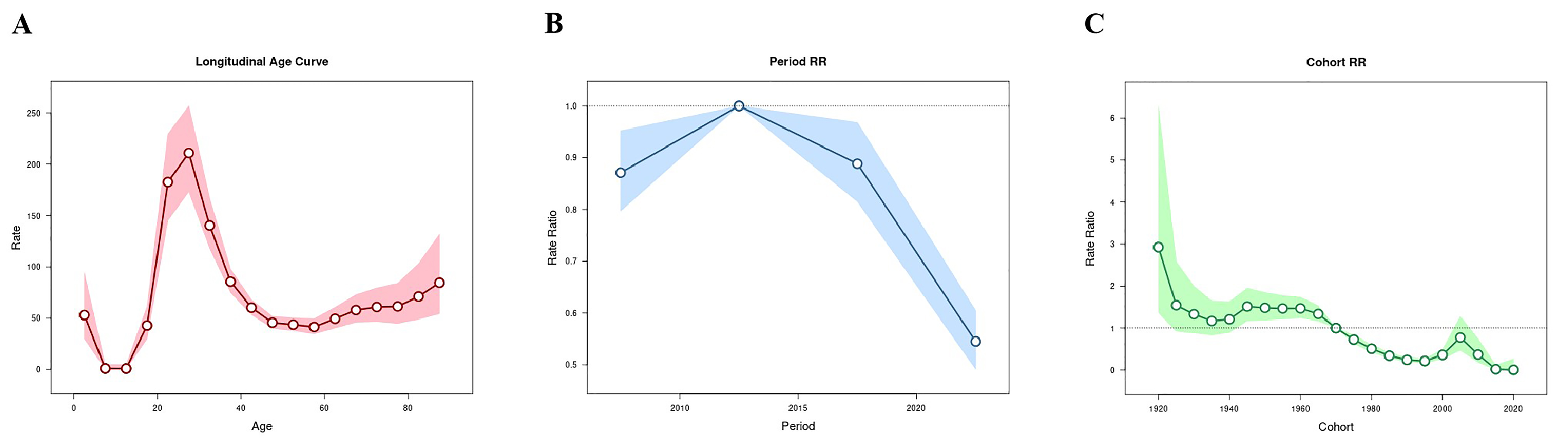

Supplement: SUPPLEMENTARY FIGURE S2 — Age-period-cohort effects on the reported incidence of syphilis in males in Zhejiang Province. [file Image_2.JPEG]

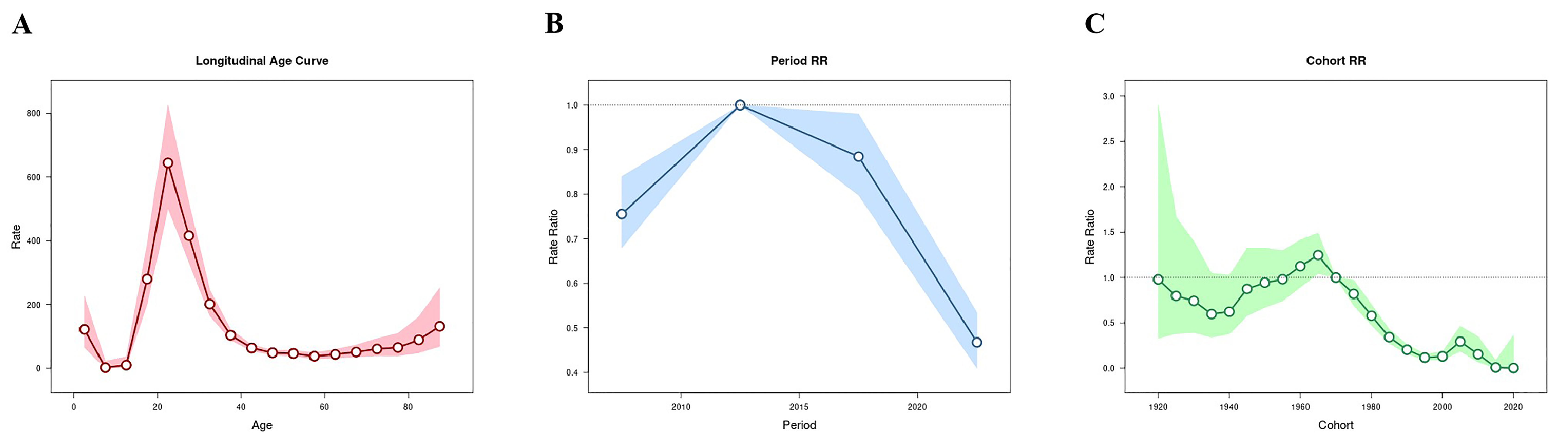

Supplement: SUPPLEMENTARY FIGURE S3 — Age-period-cohort effects on the reported incidence of syphilis in females in Zhejiang Province. [file Image_3.JPEG]
